# The Deficit Schizophrenia Subtype Is Associated with Low Adherence to the Mediterranean Diet: Findings from a Case–Control Study

**DOI:** 10.3390/jcm11030568

**Published:** 2022-01-23

**Authors:** Krzysztof Kowalski, Bogna Bogudzińska, Bartłomiej Stańczykiewicz, Patryk Piotrowski, Tomasz Bielawski, Jerzy Samochowiec, Krzysztof Szczygieł, Piotr Plichta, Błażej Misiak

**Affiliations:** 1 Department of Psychiatry, Division of Consultation Psychiatry and Neuroscience, Wroclaw Medical University, 50-367 Wroclaw, Poland; krzysztof.kowalski.umed@gmail.com (K.K.); bognaa95@gmail.com (B.B.); bartlomiej.stanczykiewicz@umed.wroc.pl (B.S.); patryk.piotrowski@umed.wroc.pl (P.P.); 2 Department and Clinic of Psychiatry, Wroclaw Medical University, 50-367 Wroclaw, Poland; tomaszbielawski90@gmail.com; 3 Department and Clinic of Psychiatry, Pomeranian Medical University, 71-457 Szczecin, Poland; samoj@pum.edu.pl (J.S.); kf.szczygiel@gmail.com (K.S.); piotrpp119@gmail.com (P.P.)

**Keywords:** nutrition, lifestyle, deficit schizophrenia, Mediterranean diet, negative symptoms

## Abstract

Accumulating evidence indicates that individuals with schizophrenia show poor dietary habits that might account for increased susceptibility to cardiovascular diseases in this population. However, it remains unknown whether this observation can be generalized over the whole population of individuals with schizophrenia. Therefore, in this study we aimed to investigate dietary habits, in terms of adherence to the Mediterranean diet (MD) in subjects with the deficit subtype of schizophrenia (SCZ-D), those with non-deficit subtype (SCZ-ND), and healthy controls (HCs). We recruited 45 individuals with SCZ-ND, 40 individuals with SCZ-D, and 60 HCs. Dietary habits were assessed using the Food Frequency Questionnaire-6 with a 12-month recall. Adherence to MD was decreased only in subjects with SCZ-D compared with HCs. Lower adherence to MD was associated with significantly higher levels of clinician-rated and self-reported negative symptoms (including alogia, avolition, and anhedonia). No significant correlations of adherence to MD with depressive symptoms were found. Lower adherence to MD was related to significantly higher body mass index in subjects with schizophrenia, but not in HCs. Our results indicate that poor adherence to MD is associated with a diagnosis of SCZ-D, higher severity of negative symptoms, and greater risk of developing overweight or obesity.

## 1. Introduction

People with schizophrenia have substantially reduced life expectancy by approximately 15 years compared with the rest of the population [1]. Apart from risky and suicidal behaviors, somatic disorders are the main contributor to this phenomenon [2]. Among somatic comorbidities, it has been found that metabolic syndrome, which consists of central obesity, atherogenic dyslipidemia, hypertension, and diabetes, is highly prevalent among individuals with schizophrenia [3]. Specific mechanisms of this observation have not been fully explained yet. Nevertheless, several lines of evidence indicate the role of intrinsic mechanisms, lifestyle factors, and antipsychotic drugs [4].

Accumulating evidence indicates that individuals with schizophrenia show poor dietary habits that can be observed even at the onset of psychosis [5]. The diet of patients with schizophrenia is generally characterized by a high intake of processed foods, saturated fats, sugar, and salt, as well as low intake of fiber, fruits, and vegetables [4]. This might also be related to a number of nutritional deficiencies observed at the early stages of psychotic disorders. Indeed, on the basis of a meta-analysis, Firth et al. found that individuals with first-episode psychosis show lower levels of folate, vitamin D, and vitamin C compared with healthy controls [6].

There is also some evidence that interventions targeting the nutritional status of individuals with schizophrenia might be beneficial in terms of improving psychopathological symptoms. For instance, it has been shown that supplementation of vitamins B might reduce psychopathological symptoms in this population [7]. More specific associations were reported for omega-3 fatty acids that have been shown to decrease the levels of positive symptoms and general psychopathology [8]. However, the association between diet and psychosis might also appear in the opposite direction. A recent study by Martland et al. demonstrated that negative symptoms might reduce responses of individuals with psychosis to lifestyle interventions targeting diet and physical activity. Altogether, these observations indicate that there is a need to investigate the dietary habits of individuals with schizophrenia with respect to psychopathological manifestation in order to personalize specific interventions [9].

It has been found that there is high interindividual variability in psychopathological manifestation of schizophrenia that might have important clinical implications. Kirkpatrick and colleagues proposed the categorization of schizophrenia diagnosis based on characteristics of negative symptoms, and they differentiated the deficit subtype (SCZ-D) [10]. A diagnosis of SCZ-D can be established when at least two primary and enduring negative symptoms are present [11]. On the basis of a meta-analysis, the prevalence of SCZ-D has been estimated at almost 33% among individuals with schizophrenia [12]. Interestingly, it has also been reported that individuals with SCZ-D show significantly higher levels of pro-inflammatory cytokines and C-reactive protein compared with subjects with non-deficit schizophrenia (SCZ-ND) or healthy controls [13]. Moreover, there is evidence that subjects with SCZ-D are at higher risk of fatal coronary heart disease and are more likely to be obese compared with individuals with SCZ-ND [8]. However, it remains unknown what are the mechanisms underlying these observations.

The Mediterranean diet (MD) is widely acknowledged to be one of the healthiest diets. It is characterized by well-balanced proportions of fatty acids, and high consumption of vegetables, fruits, nuts, and olive oil [14]. It consists of several bioactive compounds that exert neuroprotective and antioxidant activities [15]. To our knowledge, none of previous studies investigated dietary habits of individuals with SCZ-D. Therefore, in the present study, we compared dietary habits in terms of adherence to MD between individuals with SCZ-D, those with SCZ-ND, and healthy controls. Moreover, we tested the associations of adherence to MD with psychopathological manifestation of schizophrenia, cognitive performance, and body weight.

## 2. Materials and Methods

### 2.1. Participants

A total of 85 clinically stable outpatients with schizophrenia and 60 healthy controls were recruited at 2 university hospitals in Wroclaw and Szczecin (Poland) as the convenience sample. There were 40 individuals with SCZ-D and 45 individuals with SCZ-ND. Individuals with schizophrenia were enrolled if they met the following inclusion criteria: (1) age between 18 and 65 years; (2) a diagnosis of schizophrenia according to the DSM-IV criteria validated using the Operational Criteria for Psychotic Illness (OPCRIT) checklist [16]; (3) maintenance of a stable antipsychotic regimen over the period of at least 6 months; (4) symptomatic remission of positive and disorganization symptoms based on the Positive and Negative Syndrome Scale (PANSS) items (P1—delusions; P2—conceptual disorganization; P3—hallucinatory behavior; G5—mannerisms/posturing; G9—unusual thought content rated ≤ 3). The daily dosage of antipsychotics was converted to chlorpromazine equivalents (CPZeq). All groups of participants were matched for age, sex, and the level of parental education (a proxy measure of socioeconomic status).

Healthy controls had never received psychiatric diagnosis or treatment and reported no family members affected by psychotic and affective disorders in first- and second-degree relatives. In addition, a lack of psychiatric disorders was confirmed using the screening questions from the Mini International Neuropsychiatric Interview (M.I.N.I.) [17]. They were enrolled through advertisements.

The study was approved by the Bioethics Committees at Wroclaw Medical University (Wroclaw, Poland) and Pomeranian Medical University (Szczecin, Poland). All subjects gave written informed consent for participation in this study.

### 2.2. Assessment of Clinical Manifestation

All clinical assessments were performed by board-certified psychiatrists who underwent clinical training in the use of all tools administered in this study (P.P., K.S., and B.M.).

#### 2.2.1. Clinical Assessment Tools

The following measures of psychopathological manifestations were administered: (1) the Positive and Negative Syndrome Scale (PANSS) [18]; (2) the Social and Occupational Functioning Assessment Scale [19]; (3) the Calgary Depression Scale for Schizophrenia (CDSS) [20].

The Repeatable Battery for the Assessment of Neuropsychological Status (RBANS) was used to examine cognitive performance. It is composed of 12 tasks measuring 5 domains of cognitive performance: (1) immediate memory (list learning and story memory); (2) visuospatial/constructional functions (figure copy and line orientation); (3) language (picture naming and semantic fluency); (4) attention (digit span and coding); (5) delayed memory (list recall, list recognition, story memory, and figure recall) [21].

A diagnosis of the deficit schizophrenia subtype was established using the Schedule for Deficit Schizophrenia (SDS) [9]. The SDS is a semi-structured interview that records characteristics of six negative symptoms, including restricted affect, diminished emotional range, poverty of speech, curbing of interests, diminished sense of purpose, and diminished social drive. According to the SDS, a diagnosis of the deficit subtype of schizophrenia can be established in case of at least 2 negative symptoms with the following clinical characteristics: (1) primary character (i.e., not attributable to extrapyramidal side effects, depression, anxiety, or psychotic symptoms); (2) enduring presence (detectable in the preceding 12 months, including periods of clinical stability), (3) at least moderate severity (rated on a 5-point scale: 0—not present; 2—moderate; 4—very severe).

#### 2.2.2. The Self-Evaluation of Negative Symptoms (SNS)

The Polish version of SNS was administered to record self-assessment of negative symptoms [22,23]. It is a 20-item self-report evaluating 5 domains of negative symptoms, including social withdrawal, diminished emotional range, alogia, avolition, and anhedonia. Each item is based on a 3-point Likert-like scale (0—strongly disagree; 1—somewhat agree; 2—strongly agree). The total SNS score ranges between 0 and 40 with higher scores indicating greater severity of negative symptoms. The Cronbach’s alpha of the SNS in the group of individuals with schizophrenia was 0.920, indicating very good internal consistency.

### 2.3. Assessment of Adherence to the Mediterranean Diet

Dietary intake was assessed using a 62-item Food Frequency Questionnaire-6 (FFQ-6) [24]. It records food frequency consumption in the preceding 12 months. Each item measures the frequency of consuming specific food products based on a 6-point scale: 1—“never or almost never”; 2—“once a month or less”; 3—“several times a month”; 4—“several times a week”; 5—“daily”; 6—“several times a day”. Data collected using the FFQ-6 allow to assess adherence to the Mediterranean diet using the aMED score [25]. The following food categories are used to calculate the aMED score: (1) vegetables; (2) fruits; (3) whole grains; (4) fish; (5) legumes; (6) nuts and seeds; (7) the ratio of vegetable oils to animal fat; (8) red and processed meat. Participants with the intake above (or below in case of red and processed meat) the median intake among healthy controls receive 1 point; otherwise, they receive 0 points. The total aMED score ranges between 0 and 8, with higher scores representing greater adherence to the Mediterranean diet. The Cronbach’s alpha of the FFQ-6 was 0.820 in the total sample, indicating good internal consistency.

### 2.4. Statistics

Between-group differences in categorical variables were assessed using the χ^2^ tests. The Kruskal–Wallis test or one-way analysis of variance (ANOVA), depending on data distribution, were used to compare individuals with SCZ-D, SCZ-ND, and healthy controls with respect to continuous variables. The Games–Howell test (in case of one-way ANOVA) and Bonferroni correction (in case of Kruskal–Wallis test) were applied as post hoc tests. The analysis of covariance (ANCOVA) was performed to test between-group differences in the aMED score. Age, sex, and the level of education were added as covariates. Bivariate correlations of the aMED score were analyzed using the Spearman rank correlation coefficients. Significant bivariate correlations of the aMED score were further analyzed using linear regression analyses. The variance inflation factor (VIF) was applied as the measure of collinearity diagnostics [26]. The VIF > 4 was considered to indicate significant multicollinearity. The level of significance was set at *p* < 0.05 in all analyses. The Statistical Package for Social Sciences (SPSS), version 28, was used to perform data analyses.

## 3. Results

The demographic and clinical characteristics of participants are shown in Table 1. There were no significant between-group differences in age, sex, or own and parental education. Both groups of individuals with schizophrenia had similar illness duration and the severity of depressive symptoms. However, the dosage of antipsychotics was significantly higher in subjects with SCZ-D. Individuals with SCZ-D, but not those with SCZ-ND, had significantly higher BMI compared with healthy controls. There were also significant between-group differences with respect to cognitive performance, the severity of negative symptoms, and social and occupational functioning.

**Figure 1:**
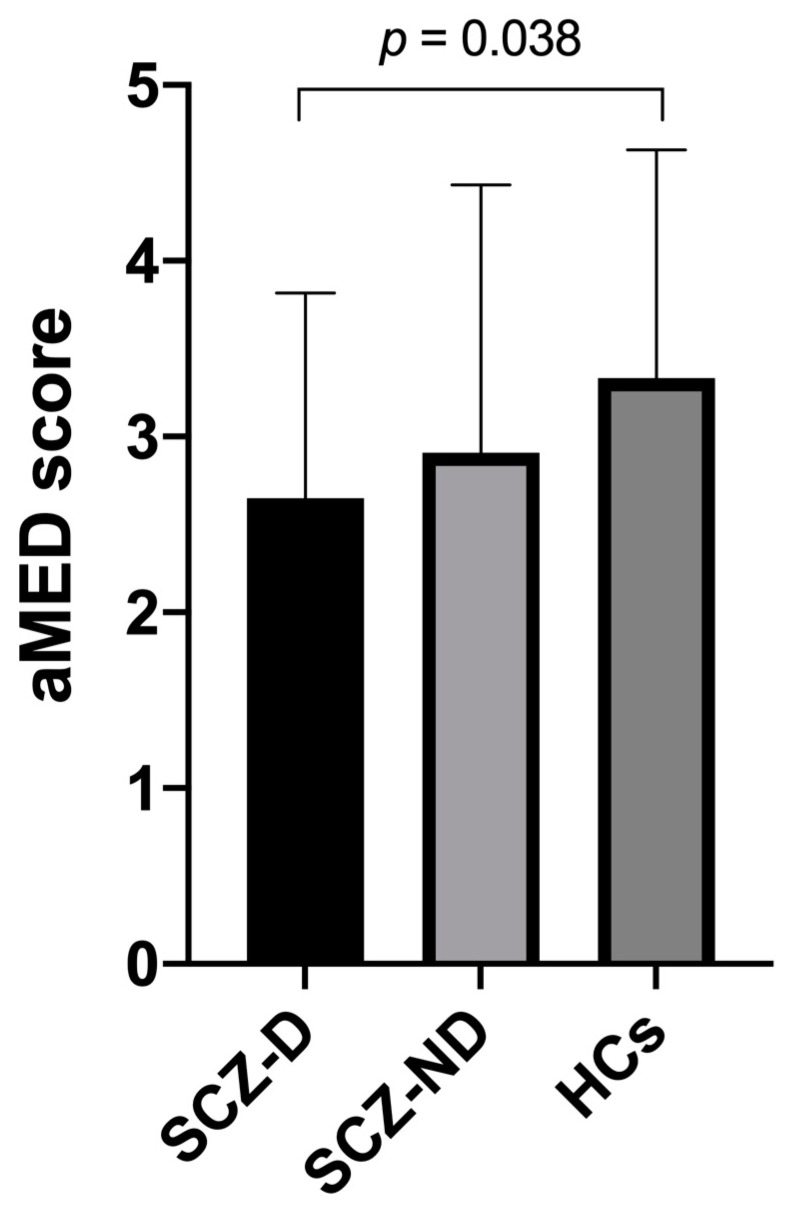
Mean aMED scores in individuals with deficit schizophrenia (SCZ-D), non-deficit schizophrenia (SCZ-ND), and healthy controls (HCs). Error bars represent standard deviations.

The aMED score was significantly lower in subjects with SCZ-D, but not in those with SCZ-ND, compared with healthy controls (Figure 1). The ANCOVA revealed that this difference remained significant (F = 3.845, *p* = 0.024) after co-varying for age (F = 0.615, *p* = 0.434), sex (F = 1.029, *p* = 0.312), and the level of education (F = 5.545, *p* = 0.020).

**Figure 2:**
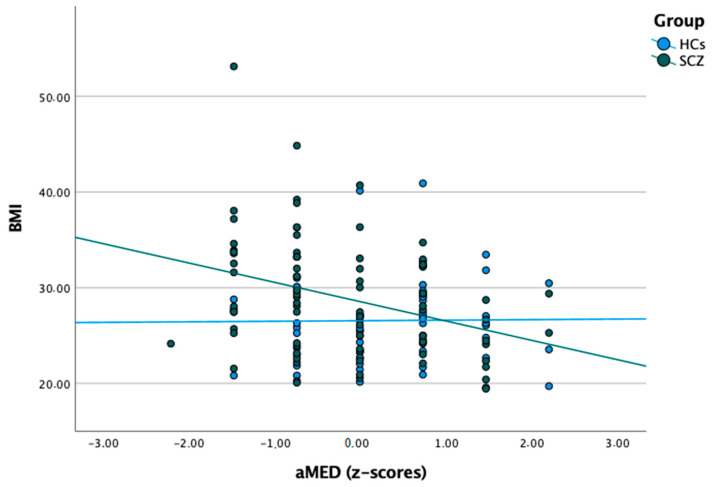
Correlations between the aMED score and BMI in subjects with schizophrenia and healthy controls.

Bivariate correlations of the aMED score among individuals with schizophrenia and healthy controls are reported in Table 2 and Figure 2. There were significant negative correlations of the aMED score with BMI, the PANSS scores of negative symptoms, and the SNS scores (total score, alogia, avolition, and anhedonia). No significant correlations of the aMED score were found in healthy controls. Due to very low SNS scores in healthy controls, correlations of the SNS score with the aMED score were not assessed in this group of participants.

Significant bivariate correlations were further tested using linear regression analysis. These analyses revealed significant effects of group (schizophrenia vs. healthy controls) and the group × aMED score interaction on BMI (Table 3, model 1). However, the effect of the group × aMED score interaction appeared to be not significant after adding age, sex, and the level of education as covariates (Table 3, model 2). Importantly, adding these covariates was not associated with a significant increase in the percentage of variance explained by the model. Indeed, model 1 explained 12.0% of variance in BMI, while model 2 explained 12.7% of variance in BMI (F change = 0.367, *p* = 0.777). Linear regression analyses also demonstrated significant correlations of the aMED scores with the PANSS scores of negative symptoms and the SNS scores (total score, alogia, avolition, and anhedonia) after adjustment for illness duration and CPZeq (Table 4).

## 4. Discussion

To the best of our knowledge, this is the first study that explored the relationship between SCZ-D and adherence to MD. We found that individuals with SCZ-D, but not those with SCZ-ND, show worse adherence to MD compared with healthy controls. The dosage of antipsychotics was significantly higher in subjects with SCZ-D, which is not in line with previous studies [27,28,29]. Despite of this fact, the correlation of the aMED score with CPZeq was not significant. To date, little is known about adherence to MD among individuals with schizophrenia. A recent study by Costa et al. revealed that both inpatients and outpatients with schizophrenia show poor-to-moderate diet quality in terms of adherence to MD. However, the authors did not differentiate their sample based on the SCZ-D diagnostic criteria, and they did not recruit healthy controls [30].

Moreover, we found that higher severity of negative symptoms (clinician-based ratings and self-assessment), especially alogia, avolition, and anhedonia, is associated with poor adherence to MD. Notably, we did not find significant associations of adherence to MD with depressive symptoms that might be closely related to negative symptoms. In agreement with our findings, Hahn et al. reported lower fruit and vegetable intake in patients with higher severity of negative symptoms [31]. Similarly, Jakobsen et al. found that low diet quality operationalized by the consumption of fish, fruits, vegetables, and fat might be associated with higher levels of negative symptoms [32]. More recently, Martland et al. revealed in their study that lower intake of fresh fruit and vegetables is related to higher severity of negative symptoms assessed by means of the PANSS at baseline [9]. However, in this study, baseline levels of negative symptoms were not associated with adherence to positive dietary changes after a 12-month intervention that had included cognitive behavioral therapy and motivational interviewing to support behavior changes in key areas of lifestyle.

Importantly, our study did not find a significant association between adherence to MD and cognitive performance, neither in subjects with schizophrenia nor in healthy controls. Although there is some evidence that better adherence to MD might be associated with better cognitive performance in non-psychiatric populations, none of previous studies aimed to address this hypothesis in subjects with schizophrenia [33]. A recent systematic review of studies investigating the efficacy of dietary interventions in schizophrenia reported 19 studies showing improvement in one or more domains, including psychopathology, cognition, and quality of life. However, the authors noticed that studies reporting positive findings with respect to cognitive performance had smaller sample size and were less likely to be randomized [34]. Moreover, it should be noted that adherence to MD represents one of several characteristics of dietary patterns, and thus we cannot conclude about the impact of other aspects of diet on cognitive performance. For instance, Adamowicz et al. found that a broader dietary intervention, including not only adherence to consumption of “healthy” products but also the elimination of sweet and regularity of food consumption, improves cognitive skills among individuals with schizophrenia [35].

It is noteworthy that adherence to MD was negatively correlated with BMI only among individuals with schizophrenia. A significant association between adherence to MD and BMI in healthy controls was not observed. However, they had significantly better adherence to MD, suggesting that the effect of poor diet quality on BMI might appear at the certain threshold. Indeed, it has been shown in non-psychiatric populations that poor adherence to MD affects the development of obesity after crossing the specific cut-off [36]. It is important to note that MD has several properties which might help to maintain appropriate weight. It contains several plant-based foods rich in fiber, which is responsible for increased satiety. Furthermore, it is characterized by low energy density and contains low glycemic load products [37]. Of note, poor adherence to MD in subjects with psychotic disorders might appear at illness onset. For instance, Saugo et al. found that most individuals (60%) with first-episode psychosis show poor adherence to MD. Moreover, the authors observed that poor adherence to MD is significantly associated with higher BMI and total cholesterol levels [38]. Similarly, Scoriels et al. identified that BMI is associated with decreased consumption of vegetables [5]. Another study also demonstrated that “healthy dietary pattern”, characterized by high consumption of fish and vegetables, is related to lower BMI in subjects with schizophrenia [39]. However, the mechanisms underlying the preference of unhealthy dietary patterns among individuals with schizophrenia have not been thoroughly addressed. One of potential mechanisms is that a brain reward circuitry plays a key role in this phenomenon. Antipsychotics might exacerbate alterations in this neural network due to their dopaminergic component [40]. Hence, individuals with schizophrenia prefer highly palatable, energy-dense foods (i.e., high-fat, sweet snacks) which provide immediate reward. Additionally, a lack of motivation, which is more pronounced in individuals with SCZ-D, might be related to less involvement in food preparation and preference of undifferentiated dietary pattern with highly processed foods [41].

Certain limitations need to be considered during the interpretation of our findings. First, our study is characterized by a low sample size. Second, the use of a convenience sample may not provide generalizability of findings over the whole population of individuals with schizophrenia. Third, due to a cross-sectional design, it is not possible to indicate causal associations. Fourth, we did not record inter-rater reliability measures. However, all clinicians involved in clinical assessment underwent a thorough training in the use of all tools administered in this study. Additionally, the living arrangement of individuals with schizophrenia and their role in diet planning was not assessed. Another limitation is that we used self-reports to assess dietary habits, and the recall bias cannot be excluded due to recording food intake over the preceding 12 months. Moreover, a relatively low percentage of variance explained by our models also suggests that there are other factors explaining the association of dietary habits with body weight and negative symptoms in subjects with schizophrenia. At this point, it is important to note that we did not record other variables, e.g., physical activity, sedentary behaviors, alcohol consumption, and cigarette smoking. Additionally, we did not analyze the effects of specific antipsychotics due to the small sample size.

In conclusion, the present findings indicate that individuals with SCZ-D show poor adherence to MD. Poor dietary habits, operationalized as adherence to MD, might be associated with the development of overweight or obesity as well as greater severity of negative symptoms. Our findings might have important implications for clinical practice and suggest that enhancing adherence to MD might improve mental and physical health outcomes in subjects with SCZ-D.

## Figures and Tables

**Table 1:** General characteristics of the sample.

	1. SCZ-D, *n* = 40	2. SCZ-ND, *n* = 45	3. HCs, *n* = 60	*p*	Significant Pairwise Comparisons
Age, years	46.3 ± 14.5	43.4 1± 2.4	44.6 ± 14.2	0.744	-
Sex, males (%)	31 (77.5)	26 (57.8)	39 (65.0)	0.153	-
Education, higher (%)	7 (17.5)	14 (31.1)	8 (13.3)	0.071	-
Mother’s education, higher (%)	7 (17.5)	8 (17.8)	7 (11.7)	0.613	-
Father’s education, higher (%)	7 (17.5)	11 (24.4)	6 (10.0)	0.141	-
BMI, kg/m^2^	29.9 ± 5.8	28.0 ± 6.4	26.6 ± 4.3	**0.013**	1 > 3
RBANS—global cognition	165.7 ± 37.8	193.7 ± 39.0	221.5 ± 23.7	**<0.001**	1 < 2, 1 < 3, 2 < 3
RBANS—immediate memory	33.7 ± 9.8	36.5 ± 7.2	46.2 ± 6.1	**<0.001**	1 < 3, 2 < 3
RBANS—visuospatial/constructional	30.4 ± 7.6	32.5 ± 6.5	37.3 ± 2.8	**<0.001**	1 < 3, 2 < 3
RBANS—language	26.5 ± 8.2	34.0 ± 16.9	30.3 ± 5.5	**0.005**	1 < 2, 1 < 3
RBANS—attention	37.3 ± 13.1	45.8 ± 14.9	56.3 ± 12.1	**<0.001**	1 < 2, 1 < 3, 2 < 3
RBANS—delayed memory	37.8 ± 10.8	44.9 ± 8.3	51.4 ± 8.1	**<0.001**	1 < 2, 1 < 3, 2 < 3
Illness duration, years	21.8 ± 12.6	16.9 ± 12.8	-	0.075	-
CPZeq, mg/day	676.9 ± 396.9	478.8 ± 278.4	-	**0.022**	
CDSS	2.3 ± 3.4	1.3 ± 1.7	-	0.490	-
PANSS—negative symptoms	17.0 ± 5.3	9.4 ± 4.7	-	**<0.001**	-
SNS—total score	23.0 ± 9.0	11.5 ± 7.3	0.3 ± 0.8	**<0.001**	1 > 2, 1 > 3, 2 > 3
SNS—social withdrawal	4.7 ± 2.7	2.2 ± 1.7	0.1 ± 0.4	**<0.001**	1 > 2, 1 > 3, 2 > 3
SNS—diminished emotional range	4.7 ± 2.2	2.3 ± 1.8	0.1 ± 0.4	**<0.001**	1 > 2, 1 > 3, 2 > 3
SNS—alogia	4.6 ± 2.6	2.4 ± 2.0	0.05 ± 0.2	**<0.001**	1 > 2, 1 > 3, 2 > 3
SNS—avolition	4.7 ± 2.4	2.6 ± 2.2	0	**<0.001**	1 > 2, 1 > 3, 2 > 3
SNS—anhedonia	4.4 ± 2.4	2.1 ± 1.8	0.03 ± 0.2	**<0.001**	1 > 2, 1 > 3, 2 > 3
SOFAS	46.8 ± 11.3	66.3 ± 19.0	-	**<0.001**	-

Significant differences (*p* < 0.05) were marked with bold characters. Data expressed as mean ± SD or *n* (% of cases). Abbreviations: BMI—body mass index; CDSS—the Calgary Depression Scale for Schizophrenia; CPZeq—chlorpromazine equivalent dosage; SOFAS—the Social and Occupational Functioning Assessment Scale; HCs—healthy controls; PANSS—the Positive and Negative Syndrome Scale; RBANS—the Repeatable Battery for the Assessment of Neuropsychological Status; SCZ-D—individuals with deficit schizophrenia; SCZ-ND—individuals with non-deficit schizophrenia; SNS—the Self-Evaluation of Negative Symptoms.

**Table 2:** Bivariate correlations of the aMED score.

	SCZ	HCs
BMI, kg/m^2^	***r* = −0.320, *p* = 0.003**	*r* = 0.034, *p* = 0.799
RBANS—global cognition	*r* = −0.058, *p* = 0.601	*r* = −0.111, *p* = 0.400
RBANS—immediate memory	*r* = −0.077, *p* = 0.485	*r* = −0.066, *p* = 0.618
RBANS—visuospatial/constructional	*r* = −0.005, *p* = 0.967	*r* = −0.218, *p* = 0.094
RBANS—language	*r* = 0.028, *p* = 0.802	*r* = 0.218, *p* = 0.094
RBANS—attention	*r* = −0.055, *p* = 0.622	*r* = −0.107, *p* = 0.416
RBANS—delayed memory	*r* = −0.109, *p* = 0.324	*r* = −0.111, *p* = 0.397
CPZeq	*r* = −0.044, *p* = 0.691	-
CDSS	*r* = −0.085, *p* = 0.443	-
PANSS—negative symptoms	***r* = −0.249, *p* = 0.022**	-
SNS—total score	***r* = −0.303, *p* = 0.005**	-
SNS—social withdrawal	*r* = −0.129, *p* = 0.243	-
SNS—diminished emotional range	*r* = −0.111, *p* = 0.316	-
SNS—alogia	***r* = −0.289, *p* = 0.008**	-
SNS—avolition	***r* = −0.363, *p* < 0.001**	-
SNS—anhedonia	***r* = −0.279, *p* = 0.010**	-
SOFAS	*r* = 0.183, *p* = 0.095	-

Significant correlations (*p* < 0.05) were marked with bold characters. Abbreviations: BMI—body mass index; CDSS—the Calgary Depression Scale for Schizophrenia; CPZeq—chlorpromazine equivalent dosage; SOFAS—the Social and Occupational Functioning Assessment Scale; HCs—healthy controls; PANSS—the Positive and Negative Syndrome Scale; RBANS—the Repeatable Battery for the Assessment of Neuropsychological Status; SCZ—individuals with schizophrenia; SNS—the Self-Evaluation of Negative Symptoms.

**Table 3:** Linear regression analysis for the association between BMI and the aMED score.

	Independent Variable	Beta	*t*	*p*	VIF
Model 1 R^2^ = 0.120 (F change = 6.334, *p* < 0.001)	Group	0.177	2.175	**0.031**	1.048
	aMED score	0.010	0.080	0.936	2.657
	Group × aMED score	−0.287	−2.256	**0.026**	2.582
Model 2 R^2^ = 0.127 (F change = 0.367, *p* = 0.777)	Group	0.187	2.264	**0.025**	1.074
	aMED score	−0.005	−0.037	0.971	1.030
	Group × aMED score	−0.249	−1.838	0.068	2.883
	Age	0.033	0.403	0.687	1.030
	Sex	−0.012	−0.148	0.883	1.022
	Education level	−0.079	−0.908	0.365	1.196

Significant associations (*p* < 0.05) were marked with bold characters.

**Table 4:** Linear regression analyses for the association between the aMED score and negative symptoms.

Dependent Variable	Independent Variable	Beta	*t*	*p*	VIF
PANSS—negative symptoms R^2^ = 0.175	aMED score	−0.311	−2.966	**0.004**	1.004
	Illness duration	0.149	1.440	0.154	1.004
	CPZeq	0.225	2.174	**0.033**	1.003
SNS—total score R^2^ = 0.205	aMED score	−0.249	−2.441	**0.017**	1.004
	Illness duration	0.266	2.615	**0.011**	1.005
	CPZeq	0.261	2.560	**0.012**	1.003
SNS—alogia R^2^ = 0.102	aMED score	−0.245	−2.263	**0.026**	1.004
	Illness duration	0.077	0.711	0.479	1.005
	CPZeq	0.180	1.663	0.100	1.003
SNS—avolition R^2^ = 0.266	aMED score	−0.317	−3.239	**0.002**	1.004
	Illness duration	0.276	2.816	**0.006**	1.005
	CPZeq	0.287	2.936	**0.004**	1.003
SNS—anhedonia R^2^ = 0.166	aMED score	−0.235	−2.255	**0.027**	1.004
	Illness duration	0.263	2.521	**0.014**	1.005
	CPZeq	0.193	1.849	0.068	1.003

Significant associations (*p* < 0.05) were marked with bold characters. Abbreviations: CPZeq—chlorpromazine equivalent dosage; PANSS—the Positive and Negative Syndrome Scale; SNS—the Self-evaluation of Negative Symptoms.

## Data Availability

Data obtained in the study will be available upon reasonable request sent to the corresponding author.
